# Bayesian ^13^C-Metabolic Flux Analysis of Parallel Tracer Experiments in Granulocytes: A Directional Shift within the Non-Oxidative Pentose Phosphate Pathway Supports Phagocytosis

**DOI:** 10.3390/metabo14010024

**Published:** 2023-12-29

**Authors:** Melanie Hogg, Eva-Maria Wolfschmitt, Ulrich Wachter, Fabian Zink, Peter Radermacher, Josef Albert Vogt

**Affiliations:** Institute for Anesthesiological Pathophysiology and Process Engineering, Ulm University Medical Center, 89081 Ulm, Germany; eva-maria.wolfschmitt@uni-ulm.de (E.-M.W.); ulrich.wachter@uni-ulm.de (U.W.); fabian.zink@uni-ulm.de (F.Z.); peter.radermacher@uni-ulm.de (P.R.); josef.vogt@uni-ulm.de (J.A.V.)

**Keywords:** Bayesian modeling, gas chromatography–mass spectrometry, glucose metabolism, immunometabolism, isotopic tracer, mass isotopomer distribution analysis, metabolic model validation, phagocytose, principal component analysis, sugar phosphates

## Abstract

The pentose phosphate pathway (PPP) plays a key role in the cellular regulation of immune function; however, little is known about the interplay of metabolic adjustments in granulocytes, especially regarding the non-oxidative PPP. For the determination of metabolic mechanisms within glucose metabolism, we propose a novel set of measures for ^13^C-metabolic flux analysis based on ex vivo parallel tracer experiments ([1,2-^13^C]glucose, [U-^13^C]glucose, [4,5,6-^13^C]glucose) and gas chromatography–mass spectrometry labeling measurements of intracellular metabolites, such as sugar phosphates and their fragments. A detailed constraint analysis showed that the permission range for net and irreversible fluxes was limited to a three-dimensional space. The overall workflow, including its Bayesian flux estimation, resulted in precise flux distributions and pairwise confidence intervals, some of which could be represented as a line due to the strength of their correlation. The principal component analysis that was enabled by these behaviors comprised three components that explained 99.6% of the data variance. It showed that phagocytic stimulation reversed the direction of non-oxidative PPP net fluxes from ribose-5-phosphate biosynthesis toward glycolytic pathways. This process was closely associated with the up-regulation of the oxidative PPP to promote the oxidative burst.

## 1. Introduction

Granulocytes are the most abundant immune cells in circulation. They play a key role in the innate immune system and are the first line of defense against pathogens [1]. At the site of infection, neutrophils ensnare microbes to kill them via oxidative burst or neutrophil extracellular trap (NETs) formation [2]. Upon stimulation, neutrophils adapt (within 10 min) their metabolic program from glycolysis, with minor oxidative PPP activity, to pentose phosphate recycling for maximization of the oxidative PPP [3]. In neutrophils, the oxidative PPP is the main source of NADPH for reactive oxygen species (ROS) production via NADPH oxidase [4,5]. Therefore, increased oxidative PPP activity after stimulation can be interpreted as a key indicator of granulocyte activation. This process is usually accompanied by a reduction to main glycolytic pathways due to competition for the substrate glucose-6-phosphate (G6P). The same effect can be caused by glycolytic mediators like TIGAR (TP53-induced glycolysis and apoptosis regulator), which decreases ROS levels by promoting PPP activity in human lymphocytes [6,7]. Substrate availability further affects the direction of the non-oxidative PPP: in a series of reversible reactions, its enzymes transaldolase and transketolase convert pentose phosphates originating from the oxidative PPP into glycolytic intermediates, including fructose-6-phosphate (F6P) and glyceraldehyde-3-phosphate (GAP). Non-oxidative PPP reactions in this direction complete the oxidative PPP and have been most widely studied, but reactions in the opposite direction are equally likely and can even occur simultaneously [8]. This flexibility could transform the non-oxidative PPP into a key element of anabolic and catabolic glucose metabolism [9]. For example, Jeon et al. highlighted that “a detailed study on the role of the non-oxidative branch of PPP in neutrophils is still needed” [10].

Additional interplay between metabolic mechanisms such as gluconeogenesis and the glycogen synthesis/degradation cycle can drastically complicate the investigation of glycolysis and the PPP in granulocytes [11]. The reversibility of reactions and the vast interactions of the network call for an elaborate method to capture the entire metabolic program. To tackle this challenge, we have employed a gas chromatography–mass spectrometry (GC−MS)-based ^13^C-metabolic flux analysis (^13^C-MFA). ^13^C-MFA is a powerful tool for flux quantification. However, increased computational and technical challenges are associated with the reversibility of fluxes and the strong interactions between PPP and glycolysis as the complexity of the network increases [12]. The accuracy and precision of glucose-centered ^13^C-MFA relies on ^13^C labeling measurements of intracellular metabolites, like sugar phosphates, whose detection is a challenging task due to low intracellular levels and the occurrence of multiple isomers. In previous studies, the labeling data from ^13^C tracer experiments were most commonly determined via liquid chromatography–mass spectrometry (LC−MS and LC−MS/MS) and recently via gas chromatography–negative chemical ionization-mass spectrometry (GC−NCI−MS) [3,11,13,14,15,16]. In addition to LC−MS being costly and accompanied by struggles with baseline separation of hexose phosphates, both LC−MS and GC−NCI−MS techniques only provide labeling information of the entire carbon skeleton, resulting in the loss of valuable positional information for flux resolution, e.g., location of label concentration. In contrast, GC−MS with electron ionization (EI) provides multiple fragments with the potential to increase information for ^13^C-MFA [17]. However, the extensive fragmentation may lead to low sensitivity for certain fragment ions, especially for high *m*/*z* values like fragment ions, containing the entire carbon skeleton of sugar phosphates. The combination of low abundance of sugar phosphates and EI-fragmentation raises the question of whether the provided measurement precision is sufficient for reliable flux determination. Therefore, strategies to combat low sensitivity and the selection of optimal ^13^C-labeled glucose tracers to provide essential labeling information are critical components for the quantification of metabolic fluxes. As the gold standard in ^13^C-MFA, different tracers can be studied in parallel incubations under comparable conditions, which allows for comprehensive validation and offers the potential to increase flux accuracy and precision [18,19,20]. However, limitations arise due to the increase in required sample material, which is particularly challenging in ex vivo ^13^C-MFA approaches. We have chosen [1,2-^13^C]glucose, [4,5,6-^13^C]glucose, and [U-^13^C]glucose for our setup to simultaneously capture the oxidative and non-oxidative PPP, gluconeogenesis, and tracer dilution. The latter was especially important to detect sources of unlabeled carbon into the PPP: either frequently considered sources, like the glycogen synthesis/degradation cycle, or more theoretical inputs into triose, pentose or sedoheptulose [11,14,21].

The combination of GC−EI−MS with parallel tracer experiments enabled the detection of various fragments, including sugar phosphates that are essential for sufficient flux resolution of glycolysis and the PPP. When adapting an MFA to Bayesian analysis, we obtain not only a single, fixed flux value but flux distributions and their common, potentially nonlinear confidence regions. This enables the easy plotting of pairwise couplings, and for a single sample, we can infer the extent to which the determination of two fluxes is coupled given the underlying data set. For differences observed when comparing two or more samples or groups, Bayesian analysis facilitates the assessment of whether these differences can be explained by determination uncertainty alone or whether there is an underlying biological effect.

We therefore used this method to unravel and quantify the interplay of key processes of glucose metabolism by revealing coordinated changes with principal component analysis (PCA). Decisive pathways within the fluxes were deduced from the stoichiometric constraint matrix provided by our flux balances in the Bayesian ^13^C-MFA, where input equals output for each metabolite pool. As input for the PCA, we utilized individual ^13^C-MFA data from granulocytes (*n* = 28) of different degrees and methods of stimulation (untreated, *E. coli* bioparticle-stimulated: a bacterial pathogen, phorbol-12-myristat-13-acetat (PMA)-treated: activation of NADPH oxidase via protein kinase C activator, PMA + diphenyleneiodonium chloride (DPI)-treated: inhibition of the NADPH-oxidase complex) and investigated whether the metabolic plasticity is limited to few enough processes that changes in the individual fluxes can be detected with the given measurement accuracy.

## 2. Materials and Methods

### 2.1. Materials

RPMI 1640 powder (without glutamine, glucose, and NaHCO_3_) was produced by Genaxxon bioscience (Ulm, Germany), and the derivatization reagents N,O-bis(trimethylsilyl)-trifluoroacetamide (BSTFA) were purchased from abcr (Karlsruhe, Germany). Pig serum was obtained from Bio-Rad Laboratories (Hercules, CA, USA) and Pancoll human solution (density of 1.119 g/mL and density of 1.077 g/mL, respectively) were purchased from PAN Biotech (Aidenbach, Germany). The pHrodo™ Green *E. coli* BioParticles™ phagocytosis kit for flow cytometry and phosphate buffer saline (PBS, without Ca^2+^, Mg^2+^) were produced by Thermo Fisher Scientific (Waltham, MA, USA). We obtained Ampuwa (aqua ad iniectabilia) and sterile 0.9% NaCl solution from Fresenius Kabi (Bad Homburg, Germany). [1,2-^13^C]glucose (99 atom% ^13^C), [4,5,6-^13^C]glucose (99.5%), and [U-^13^C]glucose (99%), [U-^13^C]glucose-D-6-phosphate disodium salt hydrate (99%) were purchased from Cambridge Isotope Laboratories (Andover, MA, USA). All other chemicals and standard substances were purchased from Sigma-Aldrich (St. Louis, MO, USA).

### 2.2. Preparation of RPMI Tracer Medium

Modified RPMI-1640 medium was prepared as follows: one aliquot of RPMI 1640 powder suitable for 1 L RPMI was completely dissolved in 900 mL Ampuwa water. A 4.766 g quantity of HEPES corresponding to a final concentration of 20 mM was added before adjusting the pH to 7.5 with 1 N NaOH. The RPMI stock solution was adjusted to 1 L with Ampuwa water, sterilized using a 100 µm sterile filter, and stored at 4 °C until the isotopic tracer experiment. For experiments involving tracer mixtures, stock RPMI medium (pH: 7.5) was supplemented with 0.1 mg/mL sodium bicarbonate, 0.6 mg/mL glutamine, 0.9 mg/mL glucose and 0.9 mg/mL of the corresponding isotopic tracer ([1,2-^13^C]glucose, [4,5,6-^13^C]glucose, [U-^13^C]glucose) or unlabeled glucose for phagocytosis assay.

### 2.3. Isolation of Granulocytes from Whole Blood

Blood collection of experimental animals was performed after obtaining approval from the University of Ulm Animal Care Committee and the Federal Authorities for Animal Research (Regierungspräsidium Tübingen; Reg.-Nr. 1559, approval 29 October 2021) and in compliance with the National Institute of Health Guidelines on the Use of Laboratory Animals and the European Union’s “Directive 2010/63/EU on the protection of animals used for scientific purposes”. Arterial blood from adult, human-sized German landrace swine was collected immediately after anesthesia and instrumentation as described in detail by Münz et al. [22]. Directly after sampling, granulocytes were isolated from the lithium–heparin blood using Ficoll density centrifugation. Briefly, blood was diluted with an equal volume of PBS, layered on two-density gradient solution (density: 1.077 g/mL, 1.119 g/mL (9:8, *v*/*v*)), and centrifuged at 800× *g* (20 min without break at room temperature (RT)). The upper layer containing Ficoll and plasma was discarded, while the lower fraction containing red blood cells and granulocytes was transferred to a new tube. Osmotic lysis was performed by adding ice-cold water, incubating for 2 min, and then stopping the reaction via the addition of 10× PBS. This step was performed three times to purify the granulocytes before counting cells in a Neubauer counting chamber. All experiments comprised 10 biological replicates. Phagocytosis assay and isotopic tracer experiments were performed within 2–3 h after blood collection.

### 2.4. Phagocytosis Assay

For stimulation, 5 × 10^6^ purified granulocytes were washed with 1 mL of ice-cold RPMI 1640 medium. Cells were resuspended in 200 µL of ice-cold RPMI containing 10% pig serum. Next, 50 µL of *E. coli* BioParticles (1 mg/mL) was added to the cell suspension and incubated for 20 min at 37 °C. After stimulation, 1 mL ice-cold phagocytosis washing buffer was added, and the cells were harvested via centrifugation (5 min, 400 g, 4 °C).

### 2.5. Isotopic Tracer Experiments

For parallel isotopic labeling experiments, 5 × 10^6^ purified granulocytes with and without prior phagocytosis assay were resuspended in 1 mL RPMI medium spiked with isotopic tracer containing [1,2-^13^C]glucose, [4,5,6-^13^C]glucose, or [U-^13^C]glucose (labeled/unlabeled, 1:1, *w*/*w*), respectively. The incubation was performed in a closed 2 mL tube at 37 °C under constant vertical rotation at 5 rpm (Trayster Digital, IKA, Staufen, Germany) for 2 h. Recently, an ex vivo ^13^C analysis of neutrophils showed that a steady state of the PPP/glycolytic fluxes was achieved between 10–30 min [3]. Another study by Kuehne et al. demonstrated that the steady state of G6P ^13^C labeling was observed within 10 min when investigating the PPP in human skin [23]. Based on these previous findings, we assumed an isotopic steady state, constant fluxes, and stable labeling patterns within 2 h. After incubation, cells were washed once with 0.9% NaCl solution and subsequently stored at −80 °C after removal of all liquid. Samples were stored for about 2 weeks until analysis.

### 2.6. Extraction of Intracellular Metabolites

The frozen pellet was resuspended in 100 µL ice-cold Ampuwa water, vortexed vigorously, and sonicated in an ice bath for 10 min. Next, 400 µL of cold acetonitrile/ methanol (1:1, *v*/*v*, −20 °C) was added to the suspension. After extraction for 10 min using an ultrasonic cleaning bath, samples were centrifuged at 14,000× *g* and 4 °C for 5 min. The supernatants from duplicates of the isotopic tracer experiments were pooled in a 1.5 mL vial. The combined extract was dried at 45 °C using the SpeedVac evaporator (Savant SPD2010 SpeedVac concentrator, Thermo Scientific, Waltham, MA, USA). The residue was kept overnight at −20 °C until derivatization and GC−MS analysis.

### 2.7. Derivatization and GC−MS Analysis of Intracellular Metabolites

The cell extracts and reference standard mix containing individual concentrations of various sugar phosphates (G6P, F6P, G1P, M6P, X5P, Ru5P, R5P, DHAP, 3PG, G3P), glucose, and lactate were analyzed as ethyloxime-trimethylsilyl derivatives (EtOx-TMS). For this approach, the dried cell extracts and five staggered levels of reference standard mix (0.006 to 6 µg) were dissolved in 50 µL of ethoxyamine hydrochloride in pyridine solution (2%, *w*/*v*). After sonication for 10 min and incubation at 60 °C for 60 min, the samples were evaporated with a gentle stream of nitrogen at 45 °C. The dried residue was dissolved in 30 µL acetonitrile and 30 µL BSTFA in an ultrasonic cleaning bath (15 min). For derivatization, the reaction mixture was heated to 60 °C for 45 min. Finally, the sample was centrifuged for 5 min at 14,000× *g* before transferring the clear liquid into a GC vial for GC−MS analysis. All samples were analyzed on a 7890A/5977B GC−MS system (Agilent, Waldbronn, Germany) equipped with a 30 m OPTIMA^®^ 1301-MS column (6% cyanopropylphenyl, 94% dimethylpolysiloxane, 0.25 mm internal diameter, 0.25 µm film thickness; Macherey-Nagel, Düren, Germany). One-µL aliquots of sample solution were injected in splitless or split mode depending on peak intensity (duplicate measurements). The mass spectrometer was operated in EI mode at 70 eV. For the determination of the mass isotopomer distribution, the MS was operated in selected ion monitoring (SIM) mode (SIM parameters and GC−MS settings are provided in Supplementary file: research_data.xlsx). Integration was performed with our in-house program: A seven parameter model was used to describe the peak shape, with both legs of the peak approaching zero. The model was fitted to a measured elution curve of all relevant masses. For a given metabolite fragment, mean and standard deviation of all peak parameters were determined from measurements of at least 5 reference samples. The peak model was then used to fit a measured elution curve for a given *m*/*z* value of a study sample with the restraint that the peak parameters remain within the range of mean and standard deviation of the reference samples. The same time window was used for reference and study samples. Since the model elution curve tended toward zero at both ends, a straight line was added to it as a baseline to better track the measured curve. The carbon mass distribution (CMD) was obtained after correction for naturally occurring isotopes like nitrogen, oxygen, hydrogen and silicon with a correction matrix approach [24,25]. The CMDs were corrected for natural abundance of ^13^C for model free evaluation, but not for the modeling-based MFA, which had natural ^13^C included in its ^13^C label definition.

### 2.8. Metabolic Network Model and Metabolic Flux Analysis

#### 2.8.1. Glossary

- C1, C2, …, C6: Designation of the position for the carbons of a metabolite.
- $c_{1},c_{2},\ldots {,c}_{Nc}$: Isolated labeling on the carbons of a fragment metabolite.
- CMD: Normalized carbon mass distribution of a fragment with the elements $r_{i}$, where i denotes the mass offset or number of simultaneously labeled carbons.
- MID: Normalized mass isotopomer distribution of a fragment without correction for naturally occurring isotopes of its elements (e.g., H, C, N, O, and Si). Values are expressed as mol%.
- N: Number of carbon labeling positions of a fragment metabolite.

#### 2.8.2. ^13^C-Positional Labeling Approach

EI−MS provides the mass distributions across different fragments of the carbon skeleton. The following section shows how to derive positional labeling from the different fragments of an analyte.

The total ^13^C enrichment (T) of an ion fragment across the carbons $c_{x}$ to $c_{y}$ is denoted as:

(1) $$T_{c_{x}-c_{y}}=\sum i\cdot r_{i}$$

where $r_{i}$ refers to an element of the CMD of the fragment of interest. Alternatively, the total ^13^C enrichment can be calculated from the sum of the isolated carbon labeling, i.e.,

(2) $$T_{c_{1}-c_{6}}=c_{1}+c_{2}+c_{3}+c_{4}+c_{5}+c_{6}$$

When observing the fractional ^13^C enrichment (F),

(3) $$F_{c_{x}-c_{y}}=\frac{1}{N}\sum i\cdot r_{i}$$

refers to the average carbon labeling of a fragment. In the situation when a molecular ion or larger fragment (e.g., C1−C6) breaks down into two smaller fragments (e.g., C1−C2 and C3−C6) we obtain:

(4) $$T_{c_{1}-c_{6}}=c_{1}+c_{2}+c_{3}+c_{4}+c_{5}+c_{6}=T_{c_{1}-c_{2}}+T_{c_{3}-c_{6}}$$

from the equations above. This equation can be arranged as

(5) $$T_{c_{1}-c_{2}}=T_{c_{1}-c_{6}}-T_{c_{3}-c_{6}}$$

Thus, if a smaller fragment is cleaved from a larger one and both the initial and one of the cleaved fragments are measurable, then the difference of their total enrichments can be used to estimate the enrichment of the second fragment being formed [26].

#### 2.8.3. Accurate Assessment of the Selected Fragments for ^13^C-MFA

GC−MS measurement accuracy was the main criterion for ^13^C-MFA fragment selection. For a given fragment, the peak area of each mass isotopomer was normalized so that all signal areas from M+0 to M+(*n*+1) summed up to 1 (100 mol%):

(6) $$m_{n}=A_{n}/\sum_{i}A$$

with

n: number of carbons
$m_{n}$: fractional abundance of each isotopomer of a fragment (M+0 to M+(*n*+1))
$A_{n}:$ peak area of an individual mass isotopomer
$\sum_{i}A$: sum of all signal areas

The corresponding theoretical MIDs were calculated with the isotope distribution calculator from Agilent (MassHunter Workstation Data Analysis Core, Version 8.0.8208.0, Agilent Technologies). The measured MID of each *m*/*z* of the fragments was compared with its theoretical value. Accuracy was assessed following Zamboni’s approach [12]. An absolute error (difference) of up to 1.5 mol% was deemed acceptable for ^13^C-MFA, whereas an absolute error up to 0.8 mol% was optimal. The results for each analyzed fragment are shown in the Supplementary file: research_data.xlsx.

#### 2.8.4. Bayesian Modeling for ^13^C-MFA

We implemented a Bayesian combined model for glycolytic and PPP fluxes using the rstan package v2.21.2 (R interface to Stan) [27,28]. It utilizes an adaptive and efficient Hamiltonian Monte Carlo sampler and provides several diagnostics to check whether the inference is reliable. Its algorithm draws random samples of unknown parameters, such as fluxes. It subsequently computes CMDs from sampled fluxes by applying a user-defined model based on the elementary metabolite units (EMU) concept [29]. If the EMU-calculated CMDs are comparable to the corresponding GC−MS measurements, the sample is collected in a Markov chain Monte Carlo (MCMC) sampling chain [30]; otherwise, it is disregarded.

The resulting sampling chain contains thousands of samples of the “true” posterior distribution, from which one can derive distributions of all estimated fluxes. The probability distribution “faithfully reports the full uncertainty due to experimental error, and any potential model data incompatibilities” [31] and relies on “all collected information about an unknown model parameter” [32]. We used a Dirichlet distribution to calculate the probability that a measured CMD can be explained by a calculated CMD. This is based on the following consideration: Molecules of a fragment are in a reservoir from which NC (number of counts) samples are drawn. A distribution is calculated from these counts by dividing the counts for each individual mass by the sum of the counts for all masses. If, for a given fixed NC, more counts are randomly drawn for one mass than expected, fewer will remain for the other masses. This overestimation of the abundance of one mass and corresponding underestimation of others inevitably results in a non-negligible correlation between the individual abundances. A Dirichlet distribution captures the resulting covariance. The variance ($\sigma^{2}$) of a distribution element with abundance $M_{i}$ is denoted as:

(7) $$\sigma^{2}=\frac{\left(M_{i}\cdot (1-M_{i}\right)}{NC+1}$$

where NC serves as the precision parameter of a Dirichlet distribution.

Based on Equation (7), the variance for each element of a distribution can be specified with a single quantity NC and its isotopic abundance. Moreover, as intuitively expected, the relative variance of a distribution element increases with decreasing abundance. However, the Dirichlet distribution only captures normalization-induced correlations, but for the mentioned urn scenario, a correlation between the distribution elements that already exists before sampling is not taken into account. Thus, the Dirichlet approach remains only an approximate minimal model for error determination.

The estimated likelihood is maximal if two conditions are met: First, the difference between predicted and measured distributions should be minimal. Second, the theoretical standard deviations of the individual distribution elements should on average equal the difference between the elements of the theoretical and calculated CMD values; the precision for each fragment has to be set accordingly. More details on the adaption of Bayesian modeling for ^13^C-MFA are available in Supplementary files (Bayes_implementation.pdf, and EMU_level.xlsx).

#### 2.8.5. Stoichiometric Flux Restraint Analysis of the Metabolic Network

For MCMC sampling, flux samples were drawn from a reservoir of possible fluxes. This reservoir was defined by the requirements that (i) fluxes must not become negative and (ii) for each metabolite pool, the sum of incoming fluxes must equal the sum of outgoing fluxes. Recent Bayesian ^13^C-MFA approaches have implemented slightly different “black box” algorithms that automatically select fluxes to fit the constraints. Considering their large models (~200 fluxes), this is a necessary step [31,32,33] but results in a limited understanding of the range in which the fluxes can move. For this reason and considering our relatively small model, we specified the possible flux ranges in detail below. We can establish flux balances for the different metabolites as:

(8) S·v=0

where S is a stoichiometry matrix with the number of rows equaling the number of pools and the number of columns equaling the number of fluxes. v contains the different fluxes. The stoichiometric equation can be divided into two components:

(9) $$S_{dependent}\cdot v_{dependent}+S_{\mathrm{independent}}\cdot v_{\mathrm{independent}}=0$$

The number and selection of dependent fluxes must be chosen so that $S_{dependent}$ is a square and therefore invertible matrix. Then, the equation above can be solved for the dependent fluxes:

(10) $$v_{dependent}=S_{dependent}^{-1}\cdot S_{\mathrm{independent}}\cdot v_{\mathrm{independent}}=M_{dependent}\cdot v_{\mathrm{independent}}$$

**Figure 1:**
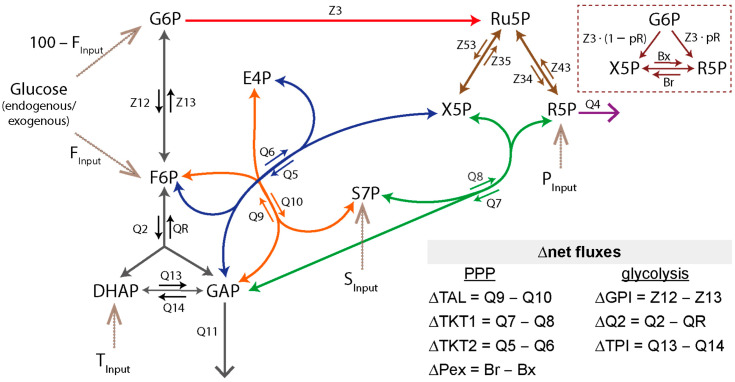
Metabolic network of the upper glucose metabolism for ^13^C-MFA. Fluxes were normalized to a glucose uptake rate of 100 into the combined F6P and G6P pool. Top right side: reduced system of the exchange between the pentose phosphate pool. Color code for fluxes: red: oxidative PPP, brown: pentose phosphate exchange, purple: R5P loss, green/blue: TKT1/2 reactions, orange: TAL reaction, black: glycolysis. The dashed lines indicate an input into the system, with P_Input_, S_Input_, and T_Input_ being unlabeled carbon sources. Abbr.: TAL: transaldolase, TKT: transketolase, P_ex_: pentose phosphate exchange, GPI: glucose-phosphate isomerase, TPI: triose-phosphate isomerase, metabolite abbr.: see Supplementary file: Bayes_implementation.pdf.

The network that we use to estimate flux rates is presented in Figure 1. In the first step, we limited the analysis to unidirectional fluxes and net fluxes for reversible reactions. Net fluxes were denoted with the prefix symbol ∆. Using Equation (10), dependent fluxes could be calculated from independent fluxes, which reduced the number of parameters. We have chosen ΔQ2, and ΔTAL as independent fluxes because they were candidates for control over the metabolic network. A third candidate for accessible control, the oxidative PPP (Z3), could not be defined as independent flux as this would lead to a non-invertible matrix in Equation (10). Thus, we selected ∆Q2, ∆TAL, and all input fluxes and arrived at Equation (11) as stoichiometric matrix.

(11) $$\left(\begin{array}{c} Z3\\ Q4\\ \begin{array}{c} Q11\\ ∆GPI\\ ∆TKT2\\ ∆TKT1\\ ∆TPI\\ ∆P_{ex} \end{array} \end{array}\right)=\left(\begin{array}{ccccccc} 1 & -1 & 2 & 0 & 0 & 0 & 0\\ 1 & -1 & -1 & 1 & 0 & 2 & 0\\ 0 & 2 & 1 & 0 & 1 & -1 & 0\\ 0 & 1 & -2 & 0 & 0 & 0 & -1\\ 0 & 0 & 1 & 0 & 0 & 0 & 0\\ 0 & 0 & 1 & 0 & 0 & -1 & 0\\ 0 & 1 & 0 & 0 & 1 & 0 & 0\\ -pX & pX & 2pR & 0 & 0 & -1 & 0 \end{array}\right)\cdot \left(\begin{array}{c} Z1\\ ∆Q2\\ \begin{array}{c} ∆TAL\\ P_{Input}\\ \begin{array}{c} T_{Input}\\ S_{Input}\\ F_{Input} \end{array} \end{array} \end{array}\right)$$

The choice of independent fluxes concluded that within the dependent fluxes, only the oxidative PPP (Z3), the R5P loss (Q4), and the glycolytic triose utilization (Q11) must be greater than or equal to zero. The resulting set of three equations derived from the first three rows of Equation (11) could thus be considered the permissible space of the constraints. These equations depended on a maximum of 6 independent fluxes when various inputs were added, corresponding to the vector on the right side of Equation (11) with Z1 (hexose input) being fixed at Z1 = 100. With independent fluxes drawn from the permissible space, the lower 5 rows of the matrix (Equation (11)) are used to calculate the net fluxes, which are not subject to any restrictions. In a final step, net fluxes must be supplemented with exchange fluxes so that forward and backward fluxes were always greater than or equal to zero. The dimension of the stoichiometric matrix generally equals the number of metabolite pools in the system (8 in the present case). By restricting the system to unidirectional and net fluxes, we could reduce the permissible flux space to three dimensions which allowed for efficient MCMC sampling. This step represents an innovation for a Bayesian MFA approach. Details are given in Supplementary file: Bayes_implementation.pdf.

In the scenario of all input fluxes being zero, we speak of a “closed glycolysis/PPP system”. For this particular case, ΔTAL and ΔQ2 were the only independently variable fluxes, resulting in only two degrees of freedom.

#### 2.8.6. Evaluation of the Bayesian ^13^C-MFA

Our ^13^C-MFA implementation contains custom code for calculating the ^13^C labeling patterns and for defining the permissible flux range. To show the correctness of the code and equivalence of results compared to established programs, we evaluated the Bayesian model against a widely used INCA-software applied by, e.g., Britt et al. [3]. For optimal comparison, we used the Britt et al. LC−MS ^13^C labeling data (3PG (C1−C3), DHAP(C1−C3), F-1,6-BP (C1−C6), 6PG (C1−C6), R5P (C1−C5), Ru5P(C1−C5), S7P(C1−C7)) as an input for our model (∑8 data sets) and disregarded Q4, P_Input_, S_Input_, and T_Input_ for model structure alignment. The following fluxes were considered in the validation: ΔGPI, ΔQ2, ΔTPI, Q11, Z3, ΔTKT1, ΔTKT2, and ΔTAL (∑8 fluxes).

#### 2.8.7. Evaluation of Flux Precision for Monitored Fragments

Crown et al. has suggested the implementation of a precision score (P) for comparison of flux precision [34]. This score $p_{i}$ can be calculated for individual fluxes:

(12) $$p_{i}={\left(\frac{{\left(97.5\%\;CI-2.5\%\;CI\right)}_{ref}}{{\left(97.5\%\;CI-2.5\%\;CI\right)}_{exp}}\right)}^{2}$$

with CI referring to the confidence interval of a flux from an MFA, utilizing only specific fragments as measurement input. These fragments can either be a reference fragment (ref, numerator) or fragments with precision scores of interests (exp, denominator). The precision score metric (P) was calculated as the average of individual flux precision scores ($p_{i}$) for n selected fluxes:

(13) $$P=\frac{1}{n}\;\sum_{i=1}^{n}p_{i}$$

We selected the following fluxes of interest: Z3, ∆TAL, ∆TKT1, ∆TKT2, Q4, Q11, ∆GPI, ∆TPI, ∆Q2, ∆P_ex_, S7P input, and H6P input (endogen) (*n* = 12). A precision score greater than one was associated with increased flux precision compared to the reference experiment.

### 2.9. Principal Component Analysis

The PCA was performed with the ^13^C-MFA results obtained from 28 samples (resting granulocytes: *n* = 10, *E. coli*-stimulated granulocytes: *n* = 10, PMA-stimulated neutrophils: *n* = 5, PMA + DPI-stimulated neutrophils: *n* = 3). The following estimated fluxes were included in this analysis: Z3, Q4, S7P input, Q11, ΔQ2, ΔTAL, ΔTKT1, ΔTKT2, and ΔGPI. The ^13^C-MFA input data were standardized to a sample mean of zero and a unit sample standard deviation of 1 before the PCA was performed in RStudio (version 2022.12.0) with the following built-in R functions: prcomp, varimax (package stats), pracma, and fviz_eig (package factoextra) [35,36]. We used the jackknife test, also called the “leave one out” procedure, for cross-validation [37,38,39]. Parameters (fluxes) were considered significant when the relative error (jackknife-SE over nominal flux value) was smaller than 0.5. Finally, to interpret the fluxes, the PCA loadings were re-scaled to the original model values.

## 3. Results and Discussion

### 3.1. GC-MS Analysis of Intracellular Metabolites as Their Ethyloxime-Trimethylsilyl Derivatives

**Figure 2:**
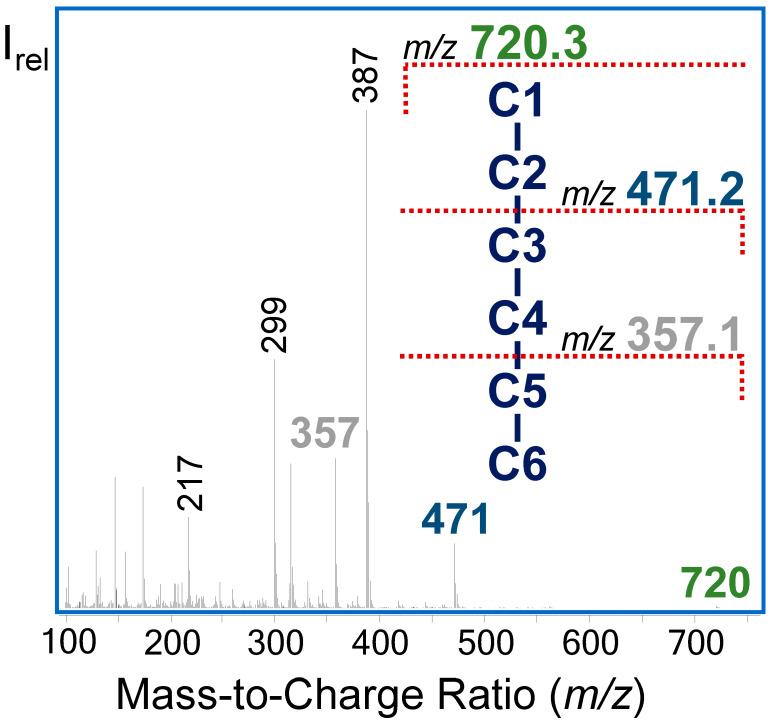
GC−EI−MS spectrum of G6P as an EtOx-TMS derivative (70 eV).

We used an ethyloxime-trimethylsilyl derivatization for determination of the ^13^C labeling pattern of intracellular metabolites. This approach allowed us to obtain multiple GC−MS fragments containing labeling information of specific parts of the molecule along with the entire carbon skeleton (Table 1, Figure 2). Moreover, we achieved baseline separation of selected metabolites like glucose, DHAP, 3PG, and hexose-phosphate isomers (G1P, F6P, M6P, G6P) within a total run time of 15 min. Pentose phosphates (P5P) such as X5P, R5P, and Ru5P could not be clearly chromatographically separated; consequently, we utilized P5P C1−C5 labeling data representing a mixture of R5P, X5P, and Ru5P. However, electron ionization allowed for the capture of specific labeling information of the lower three carbon atoms (C3−C5) of R5P. The acquired MID of the sugar phosphate fragments corresponded with the theoretical values, with an absolute error between 0.1 and 1 mol% (Supplementary file: research_data.xlsx). Monitored fragments that were deemed suitable for ^13^C-MFA are listed in Table 1.

### 3.2. Evaluation of ^13^C-Isotopomer Mass Distribution Based on Established Models and Concepts

#### 3.2.1. Characterization of Glucose Pathway Utilization Based on Interpretation of ^13^C Labeling Patterns

**Figure 3:**
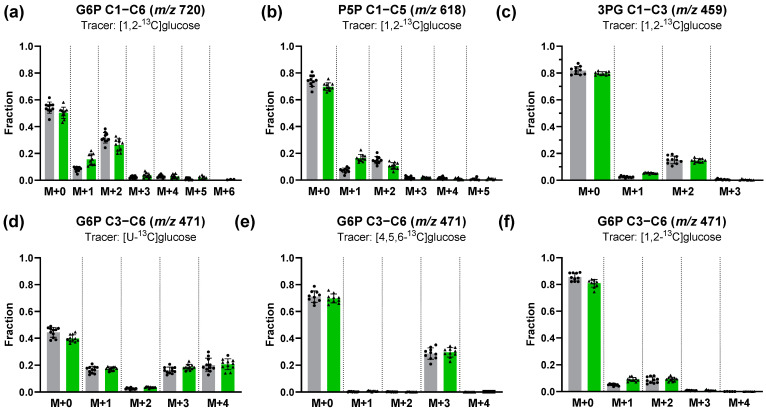
^13^C labeling patterns obtained from parallel tracer experiments with [1,2-^13^C]glucose (**a**–**c**,**f**), [U-^13^C]glucose (**d**) and [4,5,6-^13^C]glucose (**e**) of resting granulocytes (gray bars, black circle) and granulocytes after stimulation with *E. coli* bioparticles (green bars, black triangles). Mass isotopomer distributions were corrected for natural isotope abundance. Bar graphs are displayed as mean ± sd of *n* = 10 biological replicates.

**Figure 4:**
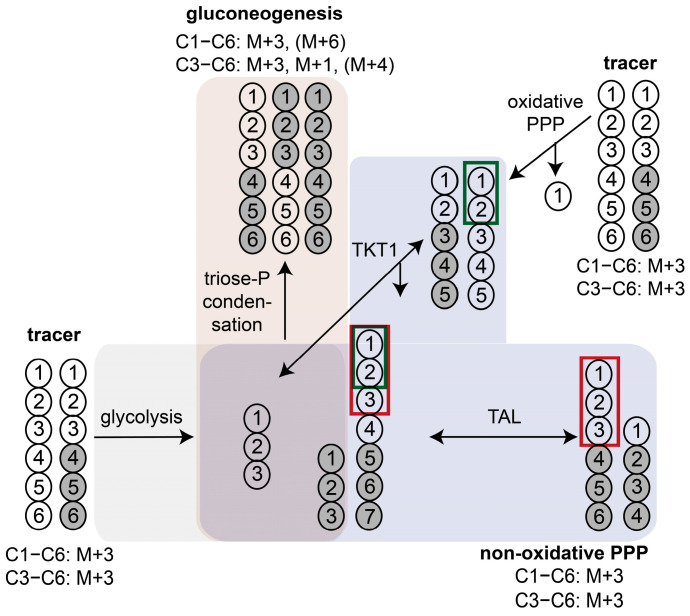
Utilization of [4,5,6-^13^C]glucose for the assessment of triose-phosphate condensation (gluconeogenesis). Metabolization of [4,5,6-^13^C]glucose via the pentose phosphate pathway produces M+3 labeling patterns on G6P (both fragments: C1−C6/C3−C6). The transaldolase/transketolase reactions only involve the upper carbon atoms (C1−C3) (shown with red and green borders), so that no ^13^C labeling is transferred from the lower half to the upper half. In contrast, triose-phosphate condensation leads to either M+3/M+6 for fragment C1–C6 or M+3/M+1/M+4 for fragment C3–C6. Due to the excess of unlabeled trioses compared to labeled trioses (3:1), the probability that both trioses are simultaneously labeled during glucose formation is low (M+4 and M+6 labeling, respectively). Abbr.: triose-P: triose-phosphate; TKT1: transketolase 1; TAL: transaldolase; PPP: pentose phosphate pathway.

Using [1,2-^13^C]glucose tracer experiments in combination with estimation of the M+1/M+2 enrichment ratio in downstream metabolites (e.g., 3PG) is the most widespread ^13^C labeling strategy used to assess relative PPP activity. Metabolism of [1,2-^13^C]glucose through glycolysis generates M+0- and M+2-labeled trioses, while metabolism via oxidative PPP produces a combination of M+0-, M+1-, and M+2-labeled intermediates. Compared to untreated granulocytes, we observed that the M+1/M+2 ratios in G6P and 3PG increased more than twofold after stimulation with *E. coli* bioparticles (Figure 3a,c), indicating that phagocytosis leads to up-regulation of the oxidative PPP relative to the glycolytic pathway. However, Antoniewicz pointed out that the equilibration of G6P and F6P affects the M+1 and M+2 ratios, limiting this strategy for relative PPP utilization to a rough estimate [40]. In this context, the notable M+1 fraction in G6P (C1−C6) provided strong evidence that PPP-derived [1-^13^C]F6P was channeled into G6P via glucose-6-phophate isomerase (GPI) and subsequently recycled into the PPP (Figure 3a). In consequence, this increased the M+0 and decreased the M+1 content, leading to an underestimation of PPP utilization. Moreover, the significant M+2 fraction in P5P (C1−C5) obtained after [1,2-^13^C]glucose tracer experiments indicated that P5P was produced via the non-oxidative PPP (Figure 3b). The [1,2-^13^C]glucose-induced M+2 and M+1 fractions in the C3−C6 fragment of G6P must have been transferred from C1 and C2 to the lower half of the hexose-6-phosphate (H6P) (Figure 3f). Additionally, the high M+1 and M+3 fractions of the G6P fragment (C3−C6, *m*/*z* 471) after [U-^13^C] tracer experiments indicated that carbons from trioses were recycled to G6P (Figure 3d). Using [U-^13^C]glucose LC−MS tracing experiments, Sadiku et al. also detected multiple isotopologues of G6P/F6P (C1−C6) in neutrophils [11]. However, interpretation of this labeling data is challenging, as PPP and glycolysis/gluconeogenesis pathways share several metabolites like GAP, F6P, and G6P, and thus the observed labeling pattern can be a result of both gluconeogenesis and the PPP. A key feature of the PPP/glycolysis-gluconeogenesis system is that all PPP reactions only exchange the first three carbons of glucose. The modified upper half can reach the triose pools of DHAP and GAP, and, in turn, labeling patterns on GAP can be incorporated into the lower half of the carbon skeleton of glucose or pentoses via the transketolase and transaldolase reactions. In a reverse reaction, labeling patterns on triose can only be incorporated into the upper half of glucose via gluconeogenesis. Accordingly, active gluconeogenesis can be demonstrated by supplying either labeled triose or lower half-labeled glucose (e.g., [4,5,6-^13^C] glucose) to the system and subsequently detecting label incorporations in the upper half of glucose (Figure 4). Following this strategy, we performed [4,5,6-^13^C]glucose tracer experiments. Here, detection of only the entire carbon skeleton of glucose is insufficient, since it is not possible to determine whether a double or triple labeling is located in the lower or upper half of the full molecule. One could try to tackle this problem by detecting higher labeling patterns that only occur via condensation of two labeled trioses (such as M+5/M+6), but this poses a challenge on its own due to the low frequency of these isotopologues. Moreover, the low abundance of the de novo synthesized patterns is exacerbated by mixing with other patterns when entering the F6P pool. It is therefore important that, in addition to the entire carbon skeleton, labeling patterns across its fragments are also recorded. Compared to the [U-^13^C]glucose tracer analysis, we only detected M+3 labeling for the G6P fragment (*m*/*z* 471) after [4,5,6-^13^C]glucose tracer experiments (Figure 3d,e). In conclusion, we can exclude the presence of gluconeogenesis based on the absence of a M+1 and M+4, indicating no transfer of lower half to upper half carbons.

#### 3.2.2. Estimation of Fractional Contribution Using the [U-^13^C]glucose Tracer

**Figure 5:**
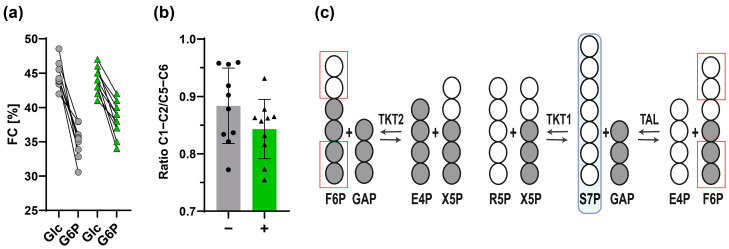
^13^C enrichment analysis of [U-^13^C]glucose tracer experiments. (**a**) Fractional contribution (FC) of glucose (Glc) (*m*/*z* 568) and G6P (*m*/*z* 720) from untreated (gray circle) and *E. coli* bioparticle-stimulated granulocytes (green triangle), *n* = 10. (**b**) Ratio of total ^13^C enrichment in C1−C2 to the total ^13^C enrichment in C5−C6 of G6P obtained from untreated (gray bars, black circle) and *E. coli*-stimulated granulocytes (green bars, black triangle). Bar graphs are displayed as mean ± sd of *n* = 10 biological replicates. The individual values are indicated by dots. (**c**) Reactions within the non-oxidative PPP: an unlabeled S7P input leads to increased ^13^C enrichment in the lower position (C5−C6) of H6P in contrast to the upper carbon atoms (C1−C2). Dark circles indicate ^13^C atoms, while empty circles represent ^12^C atoms.

The fractional contribution (FC) value resulting from fully labeled nutrients provides information about carbon sources contributing to the metabolite. In this respect, a decreased FC value may indicate incorporation of unlabeled reaction products [41]. Based on the mass isotopomer distribution, we calculated the FC as described above (Section 2.8.2). The FC was significantly lower for G6P (*m*/*z* 720, C1−C6) compared to glucose (*m*/*z* 568, C1−C6), with a greater difference in untreated granulocytes compared to activated granulocytes (Figure 5a). Both glucose uptake via glucose phosphorylation via hexokinase and dilution through the release of unlabeled carbon sources like glycogen represent potential underlying causes for the overall lower FC of G6P. In this context, the [U-^13^C]glucose tracer analysis from Ma et al. demonstrated that LPS-stimulated macrophages utilized glycogen to generate G6P as a substrate for the PPP [42]. Sadiku et al. further showed that the glycogen synthesis/degradation cycle is essential for neutrophil functions like phagocytosis [11]. Both authors reported increased tracer enrichment in glycogen and hexose-6-phosphates, combined with increased expression of glucose transporters upon activation of immune cells. In agreement with these findings, we observed a higher ^13^C enrichment of G6P upon activation of granulocytes, whereas the FC of glucose remained unaffected. Our data suggested that granulocytes increased hexokinase activity for bacterial killing, which is consistent with the finding of Ma et al. observing higher enzyme expression of hexokinase in IFN-γ/LPS-stimulated macrophages compared to untreated cells [42].

#### 3.2.3. ^13^C-Positional Labeling Analysis of G6P Indicates an Input of Unlabeled S7P into the Non-Oxidative PPP

The results of the ^13^C-MID analysis indicated that glycolysis as well as both the non-oxidative PPP and the glycogen synthesis/degradation cycle contribute to the synthesis of G6P in granulocytes. Separate labeling data in the upper and lower half of G6P generally provides information to identify potential unlabeled carbon sources. In contrast to the ^13^C dilution of G6P through glycogenolysis and glycolysis, which affects the FC of the whole molecule, incorporation of unlabeled carbon sources in the PPP causes a specific decrease of ^13^C labeling in the upper carbon atoms of G6P (Figure 5c).

We aimed to establish a GC−MS method that provides ^13^C-postional labeling information of the upper G6P carbon atoms to address this effect. However, the method did not provide a suitable fragment containing only upper half carbons (C1−C2): although we identified a fragment *m*/*z* 176 containing C1−C2, it had to be dismissed for ^13^C-MID analysis due to peak overlap with another fragment of G6P in the same *m*/*z* range. As a workaround, we calculated the ^13^C enrichment of fragment C1−C2 by subtracting the total labeling of *m*/*z* 471 (C3−C6) from *m*/*z* 720 (C1−C6) (Equation (5)). Both untreated and stimulated granulocytes displayed a lower ^13^C enrichment in the C1−C2 unit in contrast to the C5−C6 unit (*m*/*z* 357), with no significant intergroup differences (Figure 5b). These findings suggested an incorporation of unlabeled metabolites like S7P into the PPP. Interestingly, Haschemi et al. identified a seduheptulose kinase (CARKL) in macrophages, which directs the metabolic state of the non-oxidative PPP via generation of S7P from seduheptulose [14]. Another enzyme, sedoheptulose-1,7-bisphosphatase (ShB17), promoted R5P production in yeast without affecting oxidative PPP reactions by facilitating the generation of S7P from sedoheptulose-1,7-bisphosphate [43]. Through similar mediators, the transfer of the upper two carbons of unlabeled S7P to GAP could potentially result in a reduced FC value of hexose-6-phosphates, especially in C1−C2 (Figure 5c).

### 3.3. Validation and Evaluation of our Bayesian ^13^C-MFA Performance

The ^13^C labeling data of sugar phosphates resulting from parallel tracer experiments of granulocytes were applied to a ^13^C-MFA. The proposed reaction network is introduced in Figure 1. Its structure was built on the basis of the Katz/Rognstad model [44]. For comparability with the recent literature report from Britt et al., we included separate F6P and G6P compartments and an unlabeled glucose input. We also estimated 6PG and F-1,6-B patterns from the patterns of other metabolites in our system (see Supplementary file: Bayes_implementation.pdf). In addition, the above-mentioned results (Section 3.2.3) of our ^13^C-positional labeling analysis of G6P (Figure 5) indicate an input of endogenous carbon sources into the PPP. Therefore, we considered an input of unlabeled material into the P5P (P_Input_), S7P (S_Input_), and DHAP (T_Input_) pool. However, as the T_Input_ had no significant impact on the results, we disregarded it in our MFA routine.

#### 3.3.1. Validation of the Proposed ^13^C-MFA Approach: Accuracy and Precision Were Comparable with State-of-the-Art Software Packages

**Figure 6:**
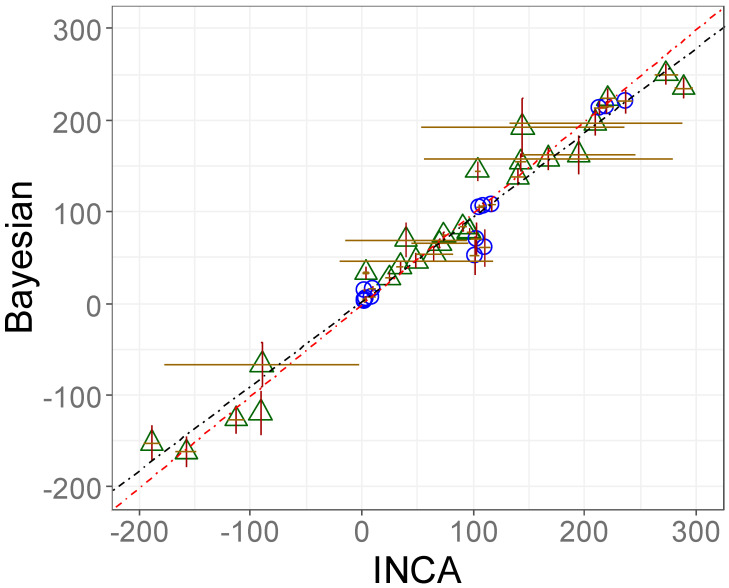
^13^C-MFA results of our Bayesian ^13^C-MFA in comparison with the ^13^C-MFA results published by Britt et al. estimated with the INCA software. Both MFA approaches utilized the same LC−MS ^13^C labeling data of Britt et al. Symbols represent mean ± sd of individual fluxes (∑8) of 8 samples (∑64 data points). Green triangles: PMA-stimulated neutrophils, blue circles: PMA + DPI-stimulated neutrophils, brown error bars: horizontal, dark red error bars: vertical, red dotted line: y = x, black dotted line: linear regression y = 0.92x + 2.78 (R^2^ = 0.958).

In contrast to a Bayesian approach, the most common software packages for ^13^C-MFA use non-linear regressions or optimization approaches based on frequentist statistics to determine the fluxes, where confidence intervals are commonly either calculated with Monte Carlo analysis or parameter continuation [45,46]. For validation of our Bayesian ^13^C-MFA model, we compared our results against a widely used INCA software that was applied by Britt et al. [3]. For this purpose, we used the same LC−MS-derived ^13^C labeling data and model structure (modified as outlined in Section 2.8.6) and achieved comparable estimated flux values and precisions (Figure 6). The marginal differences between our and Britt et al.’s results can be traced back to a different handling of the measurement error and different upper and lower bounds for the fluxes. A list of flux results with their uncertainties of each donor are available in the Supplementary file: research_data.xlsx. By showing that we achieved results very comparable to INCA’s when using the same (LC−MS) data, we could prove that the implemented Bayesian model in our study is not erroneous and follows a correct approach. However, this does not mean that additional fragment data as provided by GC−MS or the use of additional tracers would not have improved flux analysis. The advantages of multiple fragments and using parallel tracers are demonstrated in the following chapter.

#### 3.3.2. Selection of ^13^C-Labeled Glucose Tracers for ^13^C-MFA of Glycolysis and PPP

The selection of tracers for MFA is a critical step for accurate and precise flux determination [20]. To investigate whether the selected tracer of [4,5,6-^13^C]glucose, [U-^13^C]glucose, and [1,2-^13^C]glucose in combination with our proposed GC−MS analysis improved the flux resolution of our metabolic model, we tested different ^13^C-MFA scenarios. Firstly, we performed individual ^13^C-MFA for each tracer, followed by ^13^C-MFA with any combination of two tracers, and finally a complete ^13^C-MFA by applying all data sets of parallel tracer experiments to the metabolic model. For the different ^13^C-MFA scenarios, synthetic data sets were utilized to exclude the effects of potential model and measurement errors from our error analysis. Simulated data sets were generated as a random sample of different Dirichlet distributions for calculated values of a real data set.

**Figure 7:**
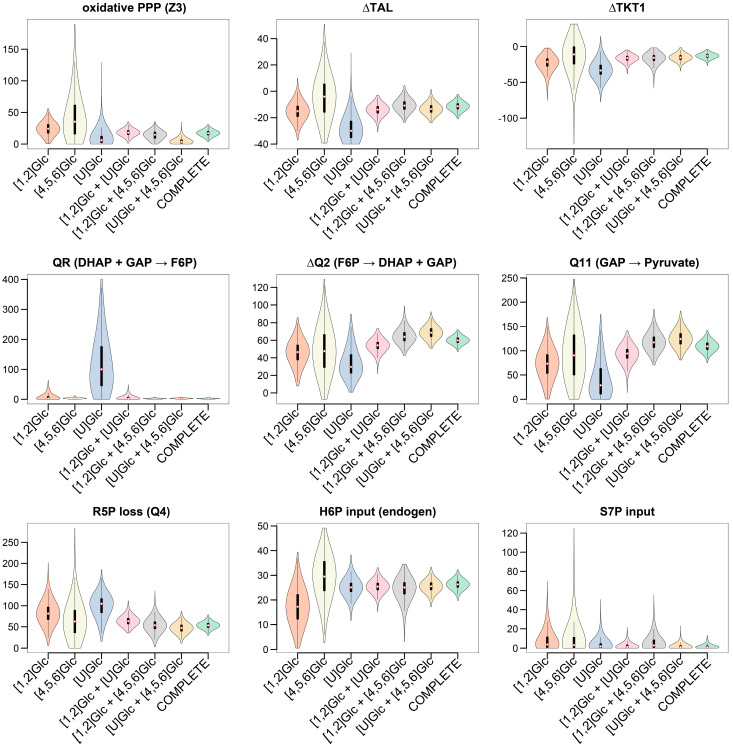
^13^C-MFA using all ^13^C labeling data (7 fragments, 4 metabolites) of individual tracer experiments and combined ^13^C-MFA of two to three tracers (complete ^13^C-MFA). Fluxes were normalized to a glucose uptake rate of 100. Results were obtained from a synthetic isotopomer data set. Violin plots represent a combination of box plot and kernel density plot. The median is highlighted as a white-filled diamond with red border. Black bars represent the interquartile range (IQR) (first and third quartile). The lower/upper adjacent values are defined as first quartile −1.5 IQR or third quartile + 1.5 IQR, respectively. They are visualized as thinner black lines extending from the IQR. The width of the violin plot reflects the frequency of data points. Oxidative PPP: Z3; non-oxidative PPP: ∆TAL, ∆TKT1, Q4 (R5P loss); condensation of triose phosphates: QR; glycolytic fluxes: ∆Q2 (triose phosphate formation), Q11 (glycolytic triose utilization).

Each tracer had unique benefits: In accordance with current literature, the [1,2-^13^C]glucose tracer was the optimal single-tracer setup to investigate the PPP (Figure 7), whereas the [U-^13^C]glucose tracer was the best performing tracer to determine tracer dilution. The [4,5,6-^13^C]glucose tracer experiment resulted in a narrow confidence interval for assessment of the condensation reaction of DHAP and GAP (QR) when compared to both the [1,2-^13^C]glucose and [U-^13^C]glucose tracers. These findings confirmed our previous assessment that the [U-^13^C]glucose tracer is not suitable for quantification of gluconeogenic pathways. Overall, parallel tracer experiments with the selected tracer combination strengthened flux information and provided partially redundant measurements, leading to a significant improvement in flux resolution of the complex glucose metabolism via glycolysis and PPP.

#### 3.3.3. GC−MS Fragments of G6P Improve Flux Precision of the Glucose Metabolism

The quality of a ^13^C-MFA depends on information-rich ^13^C patterns. Labeling data of important sugar phosphates like G6P, one of the key metabolites of glucose metabolism, often remain elusive due to challenges in MS detection, while the application of labeling data from trioses like 3PG and DHAP is relatively widespread. Recently, improvement of PPP flux resolution was achieved by utilizing ^13^C labeling data obtained from GC−MS measurements of glucose and ribose after acid hydrolysis of glycogen and RNA [47]. However, our approach has proven less time-consuming compared to this alternative approach, as sample preparation for our GC−MS analysis only required an extraction step of intracellular metabolites and the subsequent EtOx-TMS derivatization enabled the detection of several metabolites within a single GC−MS run. Compared to GC−NCI−MS and LC−MS techniques, our approach provided other fragments in addition to the fragment ion containing the intact carbon backbone of sugar phosphates, e.g., of G6P. Our final collection of fragments deemed suitable for ^13^C-MFA comprised 7 fragments of 4 intracellular metabolites. Due to the limited biological sample material, we simulated different scenarios to compare our method with alternative methods, e.g., labeling data of the entire carbon skeleton (like LC−MS/GC−NCI−MS) vs. multiple fragments. We performed the following ^13^C-MFA scenarios for the given parallel tracer experiments with [4,5,6-^13^C]glucose, [U-^13^C]glucose, and [1,2-^13^C]glucose: (i) only 3PG labeling data as input, corresponding to the most commonly used PPP determination strategy, (ii–iv): 3PG data in combination with one of the three fragments of G6P each, (v) 3PG data combined with all three G6P fragments, (vi) a combination of 3PG, DHAP and all three G6P fragments (vii) all labeling data (3PG, DHAP, G6P, P5P, ∑7 fragments). To estimate the effect of the reduced measurement precision of G6P (C1−C6) compared to the other G6P fragments (C5−C6; C3−C6), we included one additional scenario (G6P(C1−C6)* + 3PG) in which the measurement precision of G6P(C1−C6) was set to a value equivalent to other G6P fragments. The increased precision should reflect GC−NCI−MS measurement precision as soft ionization generally leads to higher signal intensity of G6P C1−C6. To simulate these scenarios, we used synthetic data sets as previously described (Section 3.3.2).

**Figure 8:**
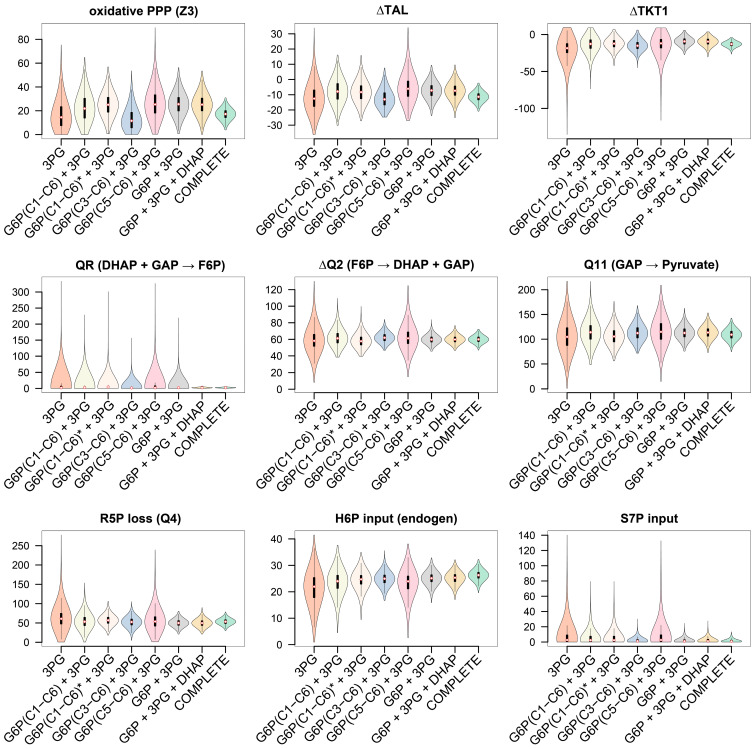
^13^C-MFA with 3PG labeling data (C1−C3) compared to (i) additional G6P labeling data of individual fragments (C1−C6, C3−C6, C5−C6). The star symbol in the set description indicates that the measurement precision of G6P(C1−C6) was set to a value equivalent to the GC−MS precision of G6P fragments C3−C6 and C5-C6. (ii) A combination of all three G6P fragments additional to 3PG. (iii) A combination of 3PG, DHAP, and all three G6P fragments, and (iv) a complete MFA (3PG, DHAP, P5P, G6P, ∑7 fragments). Results were obtained from a synthetic isotopomer data set derived from parallel tracer experiments with [4,5,6-^13^C]glucose, [U-^13^C]glucose, and [1,2-^13^C]glucose. Fluxes were normalized to a glucose uptake rate of 100. Violin plots represent a combination of box plots and kernel density plots, and the median is highlighted by a white-filled diamond with a red border. Black bars represent the interquartile range (IQR) (first and third quartile). The lower/upper adjacent values are defined as first quartile −1.5 IQR or third quartile +1.5 IQR, respectively. They are visualized as thinner black lines extending from the IQR. The width of the violin plot reflects the frequency of data points. Oxidative PPP: Z3; non-oxidative PPP: ∆TAL, ∆TKT1, Q4 (R5P loss); condensation of triose phosphates: QR; glycolytic fluxes: ∆Q2 (triose phosphate formation), Q11 (glycolytic triose utilization).

**Figure 9:**
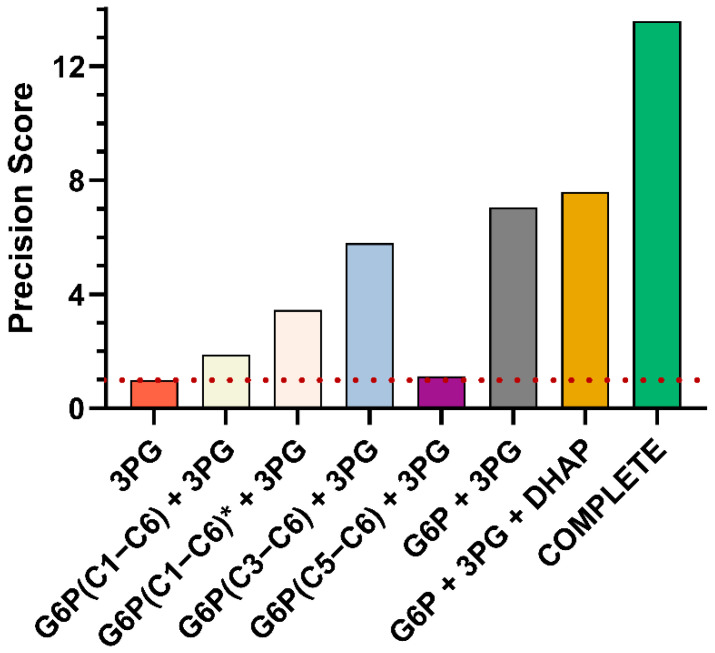
Precision scores for the given parallel tracer experiment ([1,2-^13^C]glucose, [U-^13^C]glucose, and [4,5,6-^13^C]glucose) while using different metabolites/fragments for ^13^C-MFA. The precision scores of the reference experiment (only 3PG labeling data) is 1 by definition and indicated by a red dotted line. The star symbol in the set description indicates that the reduced measurement precision of G6P (C1−C6) was set to a value equivalent to the GC−MS precision of G6P fragments C3−C6 and C5−C6.

The addition of G6P labeling information significantly increased the confidence in all flux results of glucose metabolism, with fragments of the lower half (C3−C6) being more informative compared to fragments C1−C6 and C5−C6 (Figure 8 and Figure 9). Despite the low precision of G6P (C1−C6) measurements, their inclusion into the ^13^C-MFA analysis significantly enhanced flux precision, while the G6P fragment C5−C6 only lead to a marginally improved precision when compared to using only 3PG labeling data (precision score 1.89 and 1.12, respectively). The higher precision of G6P fragment C1−C6 resulted in even narrower confidence intervals. However, the performance with all three G6P fragments increased the precision score about 2-fold compared to improvements through higher measurement precision due to simultaneous fitting of all three G6P fragments providing redundant information. Additional labeling information of DHAP combined with 3PG and G6P only slightly further improved both glycolytic and PPP flux determination, but a drastic increase in precision was observed for the condensation reaction of triose (QR). The labeling data of P5P (C1−C5, C3−C5) greatly improved flux resolution of the PPP but hardly provided any information about glycolytic fluxes like Q11 and ΔQ2.

Overall, the confidence interval of the glucose metabolism, i.e., glycolysis and PPP was improved approx. 14-fold when labeling data of the selected GC−MS fragments (G6P (C1−C6, C3−C6, C5−C6), DHAP (C1−C3), 3PG (C1−C3), and P5P (C1−C5, C3−C5)) were included in ^13^C-MFA in contrast to the reference experiment (3PG only) (Figure 9).

### 3.4. Glucose Metabolism of Granulocytes: Identification of Metabolic Patterns and Their Regulation

#### 3.4.1. The Non-Oxidative PPP Promotes Ribose-5-Phosphate Biosynthesis in Granulocytes

**Figure 10:**
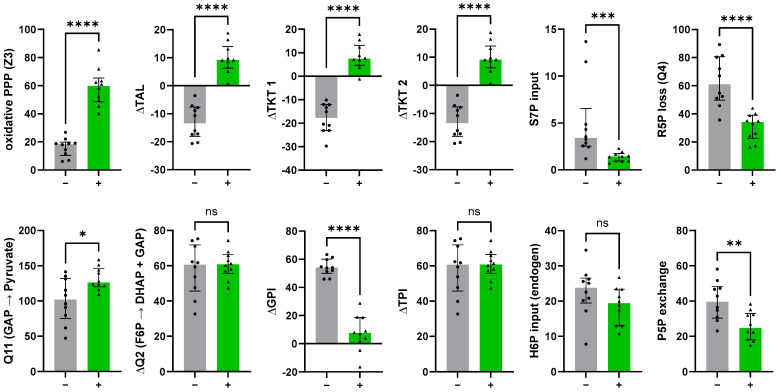
^13^C-MFA results of untreated granulocytes (−) (gray bars, black circles) and granulocytes stimulated with *E. coli* bioparticles (+) (green bars, black triangles). Fluxes were normalized to a glucose uptake rate of 100. Bar graphs show median with interquartile range of each group (*n* = 10). Individual symbols show posterior mean from Bayesian ^13^C-MFA. Statistical analysis was performed with the Mann–Whitney test (**** = *p* < 0.0001, *** = *p* = 0.0003, ** = *p* = 0.0089, * = *p* = 0.0232). Oxidative PPP: Z3; non-oxidative PPP: ∆TAL, ∆TKT1, Q4 (R5P loss), pentose phosphate (P5P) exchange; glycolytic fluxes: ∆Q2 (triose phosphate formation), Q11 (glycolytic triose utilization), ∆GPI, ∆TPI.

The ^13^C-MFA results showed that stimulation with *E. coli* bioparticles increased the oxidative PPP step (Z3) approximately 4-fold (Figure 10). In addition, the equilibrium between F6P and G6P was shifted toward G6P to enhance the oxidative PPP (Z3) to meet NADPH demand. This latter observation agrees at least partially with the reports by Britt et al. observing a reversed GPI net flux during oxidative burst of neutrophils [3]. These findings support our previously mentioned assumption that the Lee model leads to underestimation of relative PPP activity (Section 3.2.1). Moreover, the phagocytic process in granulocytes significantly shifted the transaldolase/transketolase reaction toward glucose catabolism and increased glycolytic triose utilization (Q11). The constant ∆TPI flux suggests that PPP-derived GAP was channeled toward the glycolytic pathway. Interestingly, we found an S7P input under resting conditions, suggesting that uptake of unlabeled S7P into the non-oxidative PPP promotes R5P production for nucleotide biosynthesis (Q4). The latter combined with the S7P uptake decreased significantly upon stimulation, indicating a shift away from R5P release. Our results therefore underscore the presence of a novel mechanism impacting glucose metabolism through the release of carbon sources into the non-oxidative PPP, adding further weight to the hypotheses proposed by Haschemi and others [14,48,49].

#### 3.4.2. A “Hard-Wired” PPP Network Gives Insight into Cellular Mechanisms

In addition to our GC−MS labeling data obtained after parallel tracer experiments with resting granulocytes (*n* = 10) and *E. coli*-stimulated granulocytes (*n* = 10), we utilized the LC−MS data of Britt et al. (PMA-stimulated neutrophils (*n* = 5) and PMA + DPI-stimulated neutrophils (*n* = 3)) for our Bayesian ^13^C-MFA routine [3]. The labeling data of Britt et al. enriched our data set, since it refers to a controlled, intensive stimulation of granulocytes. The metabolic data of a different donor and after a different kind of stimulation helped to cover the full metabolic plasticity of granulocytes within the glucose metabolism.

**Figure 11:**
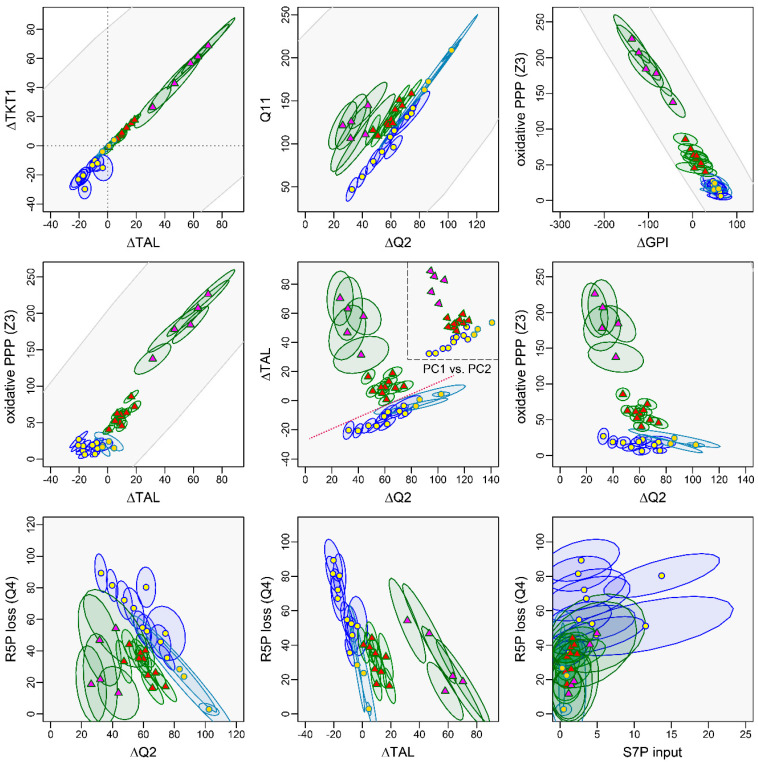
Relations between fluxes of the upper glucose metabolism. Results of the ^13^C-MFA are displayed as an ellipse with their 68% confidence interval (±one standard deviation of the mean). Dark blue ellipses with yellow circles indicate untreated granulocytes (*n* = 10), green ellipses with red triangles granulocytes stimulated with *E. coli* bioparticles (*n* = 10), light blue ellipses with beige circles PMA + DPI-stimulated neutrophils (*n* = 3), and green ellipses with magenta triangles PMA-stimulated neutrophils (*n* = 5). Symbols indicate the posterior mean of ^13^C-MFA data sets. ^13^C-MFA of granulocytes was performed with ^13^C labeling data obtained from parallel tracer experiments and subsequent GC−MS measurements. Neutrophil data were obtained by applying our ^13^C-MFA routine to LC−MS data from the recent publication of Britt et al. The gray area represents the valid range based on the model structure without fitting of ^13^C labeling data. In the center graph at the top right, we added the PCA score plot of the first two principal components for comparison. The red dotted line indicates the separation between stimulated granulocytes and non-activated granulocytes. Oxidative PPP: Z3; non-oxidative PPP: ∆TAL, ∆TKT1, Q4 (R5P loss); glycolytic fluxes: ∆Q2 (triose phosphate formation), Q11 (glycolytic triose utilization).

The main advantage of our Bayesian approach lies in the detection of joint distributions between two fluxes, e.g., via visualization of the pairwise confidence ranges. Here, a 68% confidence interval (CI) represents a 68% probability that the observed interval contains the true value [32]. We could verify that the GC−MS based ^13^C-MFA results had narrower 68% CI compared to results obtained from Britt et al.’s LC−MS data. This can be attributed to the utilization of multiple fragments and tracer experiments (Figure 7 and Figure 8). However, despite the greater measurement uncertainties, pairwise confidence regions of PMA-stimulated and *E. coli*-simulated granulocytes did not overlap. This means that we could clearly distinguish between both methods of stimulation (Figure 11, e.g., oxidative PPP vs. ΔTAL/ΔQ2). When analyzing the respective ^13^C-MFA results, we observed a strong inverse relation between ∆GPI and the oxidative PPP (Z3) as well as a direct relation between ∆TAL and ∆TKT1 (Figure 11). This was independent of granulocyte stimulation. Intriguingly, the relation between (i) the net glycolytic fluxes ∆Q2 and Q11, (ii) the R5P loss (Q4) and ∆Q2, and (iii) ∆Q2 and ∆TAL were lost once neutrophils/granulocytes became stimulated. In contrast, we observed a relation between the oxidative PPP (Z3) and ∆TAL upon immune cell activation. The frequently observed linear behavior between several fluxes could be partially explained by the stoichiometric restraint matrix (Equation (11)): 8 fluxes were calculated with respect to 4–6 independent fluxes (depending on which input fluxes were included), while Z1 was set to a fixed value of 100. Furthermore, the second and third column of the dependency matrix indicated that both ∆TAL and the glycolytic flux (∆Q2) were closely linked to all other fluxes via constraints.

**Figure 12:**
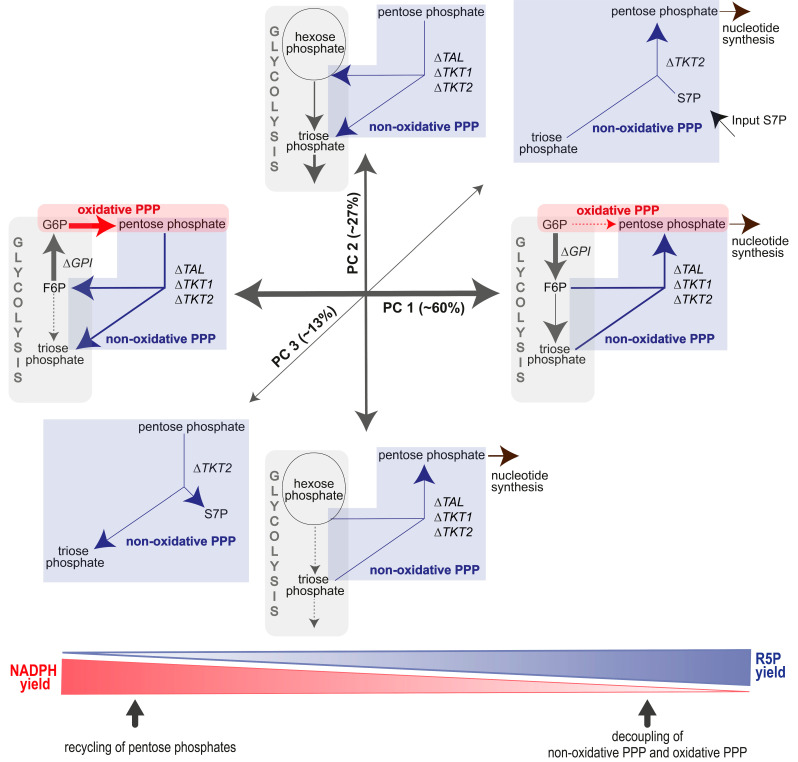
Metabolic states of granulocytes/neutrophils identified by the first three principal components (PC) of PCA exploring ^13^C-MFA data (*n* = 28, ∑_fluxes_ = 9). Contribution of each PC to the total variance indicated within parenthesis. The first three PCs amounted to ~99.6% of the variance.

Correlations between parameters can best be investigated with a PCA, which captures similar behaviors across several variables and thus allows for the detection of different directions of change. Interestingly, the PCA of the ^13^C-MFA results showed that three linear combinations of variables explain 99.6% of the variation in the data, with 86.7% of the variance being found in the first two principal component (PC1/PC2) axes. Figure 12 gives an overview of the first three PCs and their different regulatory processes (Figure 12, Table 2). PC1 refers to the directional shift in the PPP from quiescence to immune cell activation. In the positive direction, the flux from F6P to G6P increased together with the oxidative PPP. The resulting enhanced R5P production promoted the recycling of R5P to F6P and GAP instead of biosynthesis via the R5P loss of our system by either inhibiting the further use of R5P (e.g., for biosynthesis) or by an active shift in the reaction balance of transketolases. In addition, the GAP production from F6P was reduced. Analogously to results from Britt et al., the intense increase in the oxidative PPP suggests a stimulation of GPI that shifts the equilibration toward G6P and/or a inhibition of glycolytic triose formation that leads to a backlog of hexose-phosphates [3]. In summary, PC1 refers to the coupling of both PPP branches with the reversible glycolytic steps (ΔGPI, ΔQ2) to promote pentose cycling for maximization of the oxidative PPP or, in the negative direction, to the promotion of R5P synthesis via inhibition of the oxidative PPP. Neither mechanism affected pyruvate/lactate production. The second component indicated a linkage of non-oxidative PPP fluxes and downstream glycolytic fluxes to either maximize glycolytic pathways for energy supply or enhance R5P production for biosynthesis. This mechanism manifested in a positive net flux from F6P to R5P and a corresponding increase in R5P release. Furthermore, PC2 was completely independent of the oxidative PPP. This behavior could be explained by a regulatory shift of the non-oxidative PPP equilibrium toward R5P release. The associated consumption of glucose lead to a decrease in glycolytic triose and lactate production. The spread of data along the PCs 1 and 2 suggests a cell type and stimulation-specific degree of pentose phosphate production required for cell growth and function. In contrast, PC3 refers to the presence of cells in the sample that incorporated non-PPP-derived S7P into the non-oxidative arm of the PPP to promote pentose phosphate production.

When the main components PC1 and PC2 were plotted against each other, it is intriguing that they mirrored the scatterplot between ΔTAL and ∆Q2, suggesting that these key fluxes may regulate the glucose metabolism of granulocytes (Figure 11). However, it remains an open question whether these changes in ΔTAL were directly caused (as a regulatory target) or simply passive responses to other regulatory processes. The stimulated and non-stimulated cells could be clearly separated with a line when assigning PMA + DPI-treated neutrophils to the unstimulated group (Figure 11), as the confidence regions for the flux determinations of the individual cells did not protrude into the other assignment region. The status of a single cell could thus be determined unambiguously.

## 4. Conclusions

In this work, we present a novel variation of Bayesian ^13^C-MFA for precise quantification of the PPP and glycolytic fluxes, and elucidation of their metabolic interplay. The advantages of our novel method compared to alternative approaches are elaborated in Table 3. The 19 individual fluxes included in our glycolysis/PPP model could not independently assume all possible values, as flux equilibrium had to be valid for each metabolite. A theoretical analysis via a stoichiometry restraint matrix showed that its permissible space could be reduced to three dimensions. This, on the one hand, allowed for an efficient and robust MCMC sampling for the Bayesian analysis and on the other hand explained why the movement of all fluxes was limited to a three-dimensional subspace. In this study, we propose a new practical GC−MS method comprising determination of ^13^C labeling patterns of various metabolic fragments including sugar phosphates in combination with parallel tracer experiments ([1,2-^13^C]glucose, [U-^13^C]glucose, and [4,5,6-^13^C]glucose). Improved flux resolution was demonstrated via the Bayesian MFA that provided the full probability distribution of estimated fluxes which reflected the measurement uncertainties. The pairwise confidence ranges, e.g., of the net transaldolase (ΔTAL) vs. net glycolytic flux (ΔQ2), allowed for the detection of subtle differences in metabolic fluxes due to different methods of granulocyte stimulation. Estimated fluxes showed strong correlation among themselves, with individual values moving on a single line in several cases. These frequently observed behaviors enabled a principal component analysis that detected three distinct axes of coordinated flux changes that were sufficient to explain all observations in agreement with our theoretical analysis. Stimulation of granulocytes can be explained by the following mechanisms: Activation of the oxidative PPP and inhibition of glycolytic F6P degradation to trioses occur in parallel. These steps are supported by inhibition of R5P release for biosynthesis, leading to enhanced carbon rearrangement of the non-oxidative PPP to form F6P for recycling into the oxidative PPP. Granulocytes in the resting state had enhanced R5P production, which can be explained by a regulatory shift of the non-oxidative equilibrium toward R5P. Metabolic changes of all analyzed cell experiments followed these behavioral/metabolic patterns irrespective of their cell population, stimulation intensity, and cell isolation method.

## Figures and Tables

**Table 1:** Selected GC−MS fragments as EtOx-TMS derivatives for ^13^C-MFA.

Metabolite	*m*/*z*	Carbon Atoms	Molecular Formula
Glucose	568	C1−C6	C_22_H_54_O_6_NSi_5_
	319	C3−C6	C_13_H_31_O_3_Si_3_
G6P	720	C1−C6	C_25_H_63_O_9_NSi_6_P
	471	C3−C6	C_16_H_40_O_6_Si_4_P
	357	C5−C6	C_11_H_30_O_5_Si_3_P
X5P, Ru5P, R5P	618	C1−C5	C_21_H_53_O_8_NSi_5_P
R5P	459	C3−C5	C_15_H_40_O_6_Si_4_P
DHAP	414	C1−C3	C_13_H_33_O_6_NSi_3_P
3PG	459	C1−C3	C_14_H_36_O_7_Si_4_P

**Table 2:** Metabolic fluxes that contributed significantly to the first three principal components (PC) of the PCA. Contribution of each PC to the total variance is indicated within parenthesis. The first three PCs amount to ~99.6% of the variance. The sign indicates the direction of the PC relative to the mean.

Metabolic Flux		PC1 (60%)	PC2 (27%)	PC3 (13%)
**glycolysis**	∆GPI	−57.2		-6.6
	∆Q2	−10.0	−15.0	
	Q11		−32.7	
**oxidative PPP**	Z3	63.1		
**non-oxidative** **PPP**	∆TAL	24.4	−3.2	
	∆TKT1	24.8	−3.8	5.2
	∆TKT2	24.4	−3.2	
	Input S7P			−3.0
	R5P loss (Q4)	−10.1	17.7	−8.0

**Table 3:** Advantages of our proposed Bayesian ^13^C-metabolic flux analysis (MFA) method compared to alternative methods. Abbr.: EtOX-TMS: ethyloxime-trimethylsilyl derivative; TBDMS: tert-butyldimethylsilyl derivative; H6P: hexose-6-phosphate; MCMC: Markov chain Monte Carlo; NCI: negative chemical ionization; MS: mass spectrometry; GC: gas chromatography; LC: liquid chromatography.

	Novel GC−MS-Based Bayesian ^13^C-MFA	Alternative Methods	Advantage of Our Proposed Method
*Extraction and Derivatization for GC−MS analysis*	water/methanol/acetonitrile extraction one derivatization: glucose/sugar phosphates, incl. triose-phosphates (EtOX-TMS)	methanol/chloroform/water extraction [47] acid hydrolysis of glycogen and RNA [47] two derivatizations: sugar (aldonitrile propionate), triose phosphates (TBDMS) [47]	- replacement of a hazardous solvent - less time-consuming - direct labeling information of P5P/G6P for capturing dynamic processes - **efficiency/accuracy**
*Chromatography*	baseline separation H6P with a total run time of 15 min	baseline separation H6P with a total run time of 31 min (GC−NCI−MS) [16] coelution of H6P without derivatization (LC−MS) [3]	- **efficiency** - **accuracy**
*MS*	detection of multiple fragments of sugar phosphates and glucose (GC−MS)	detection of the entire carbon skeleton of sugar phosphates (LC−MS/GC−NCI−MS) [3,11,16]	- improved flux observation - redundant information - **accuracy/precision**
*Tracer experiments*	parallel tracer experiments	single-tracer experiment [3]	- improved flux observation - redundant information - **accuracy/precision**
*Formal analysis*	flux constraint analysis as a central element analysis of the feasible flux space	“black box” or integrated into software algorithms (INCA software, Bayesian MFA) [31,46,50]	- indication of permissible space: valuable information regarding cell regulation and flux relations - efficient MCMC sampling - **comprehension/efficiency**
*Bayesian ^13^C-MFA* vs. *optimization approach*	detection of multiple optima that could be caused by measurement errors	single set of flux values that best fit the measurement data	- **accuracy**
	full uncertainty distribution of fluxes	only upper and lower confidence interval	- determination of both Gaussian and non-Gaussian distributions - **comprehension/accuracy**
	pairwise correlation of fluxes with their joint confidence regions	may not be integrated into well-established MFA software (i.e., INCA)	- valuable information, whether correlation is due to measurement uncertainty or reflects a biological effect.

## Data Availability

All row data of this article are available in the Supplementary materials.

## Supplementary Materials

The following supporting information can be downloaded at: https://www.mdpi.com/article/10.3390/metabo14010024/s1. Excel file: *EMU_level.xlsx*; The EMU approach calculates labeling on metabolites in successive levels, starting with labeling of isolated carbons and ending with the highest possible number of carbons on metabolites. Each level contains a linear system of equations with an inhomogeneous vector capturing input from outside and condensation products of labeled metabolite segments calculated in lower levels. This Excel file contains the matrices and input terms for levels 1 to 4. PDF file: *Bayes_implementation.pdf*; This document introduces essential steps to implement the PPP/glycolysis model in the Stan Statistical Programming Language including: (*i*) Definitions of appropriate priors such that sampling of parameters always yields non-negative fluxes. They are subject only to the constraint that a flux balance of zero results for each metabolite. (*ii*) A simple model for the measurement error of ^13^C isotopomer distribution elements which provides error coupling between the individual distribution elements. (*iii*) A reduction in the size of the network for efficient computation without introducing any bias in the calculated labeling. Excel file: *research_data.xlsx*; This document provides the method parameters and data sets of our study: (*i*) GC−MS settings, including SIM parameters; (*ii*) model parameters and their priors; (*iii*) measured ^13^C labeling patterns of granulocytes; (*iv*) determined measurement errors of the ^13^C labeling patterns; and (*v*) flux results with the model-calculated labeling patterns and LogLik values.
